# Novel estimation technique for the carrier-to-noise ratio of wireless medical telemetry using software-defined radio with machine-learning

**DOI:** 10.1038/s41598-023-31225-3

**Published:** 2023-03-13

**Authors:** Ishida Kai

**Affiliations:** Department of Medical Engineering, Faculty of Health Sciences, Junshin Gakuen University, 1-1-1 Chikushigaoka, Minami-ku, Fukuoka, Fukuoka 815-8510 Japan

**Keywords:** Health occupations, Medical research, Risk factors

## Abstract

In this study, we developed a novel machine-learning model to estimate the carrier-to-noise ratio (CNR) of wireless medical telemetry (WMT) using time-domain waveform data measured by a low-cost software-defined radio. With automatic estimation of CNR, the management of the electromagnetic environment of WMT can be made easier. Therefore, we proposed a machine-learning method for estimating CNR. According to the performance evaluation results by 5-segment cross-validation on 704 types of measured data, CNR was estimated with 99.5% R-square and 0.844 dB mean absolute error using a gradient boosting regression tree. The gradient boosting decision tree classifiers predicted if the CNR exceeded 30 dB with 99.5% accuracy. The proposed method is effective for investigating electromagnetic environments in clinical settings.

## Introduction

Wireless medical telemetry (WMT) is a typical medical device that continuously monitors a patient’s physiological signs, such as heart rate. As WMT uses wireless communications, seamless communication is needed for patient monitoring. According to Electromagnetic Compatibility Conference Japan (EMCC) survey in 2021, about 80% of hospitals have introduced WMT, of which 60% are managing electromagnetic environments^1^.

Notably, managing WMT frequency band is necessary in establishing a good WMT reception^2^. For measuring the electromagnetic environment, we can detect poor receptions, electromagnetic interference (EMI) caused by various kinds of electrical devices, and malfunction of radio-receiving equipment to evaluate the electromagnetic environment^3^. A carrier-to-noise ratio (CNR), defined as the ratio of the received modulated carrier signal power C to the received noise power N after the receiver filters, is a criterion for the communication quality of WMT. A CNR of at least 15–20 dB is required for the minimum reception of WMT^4^. To achieve good reception, the manufacturer recommended a CNR of more than 30 dB^5^. The CNR is obtained by subtracting the background noise (BGN) power from the WMT signal power in its usage frequency. The degradation of WMT signal power may occur either by locating the transmitter at a null point or far away from the receiving antenna or the aging degradation of radio frequency components, which include receiving antennas, coaxial cables, connectors, and amplifiers^6^. However, electrical devices, especially switched-mode power supplies installed in light-emitting diode (LED) devices, may radiate unwanted emissions and increase BGN^7^. In addition, to promote the safe introduction and operation of WMT, it is necessary to consider effective frequency allocations, range of access, invasive radio waves from out-of-hospitals, and interference by intermodulation^8–10^. Other wireless communication systems, such as real-time detection systems for dementia, may interfere with WMT because these devices use the same frequency band of WMT and are widely introduced in hospitals^11^.

However, it is difficult to determine only BGN because hospitals generally use WMT transmitters for patient monitoring, whose power are hard to put off. Therefore, actual CNR is hard to measure in clinical settings. Unfortunately, there is a lack of interest in the electromagnetic compatibility (EMC) of WMT in most hospitals. Over 50% of hospitals do not have planned countermeasures for EMI, even with 20% of them having experienced EMI troubles with WMT^1^. However, only 10% of hospitals have conducted periodic quantitative investigations of WMT because only a few staff in most hospitals have adequate skills or experience regarding EMC. Moreover, it is challenging to introduce a specific measuring device for the evaluation of EMC, such as a spectrum analyzer, in most hospitals because of its cost. A simplified spectrum analysis function was installed in a WMT receiver, which measures the amplitude of received signals and/or electromagnetic noise in each WMT frequency channel^12^. However, this function cannot operate during patient monitoring. Therefore, it is desirable to establish a novel, easier, and cost-effective technique for evaluating EMC in clinical settings.

In this study, we investigated a machine-learning technique to estimate the CNR of WMT to establish a novel simplified, automated, and low-cost evaluation method.

## Concept of our approach

**Figure 1:**
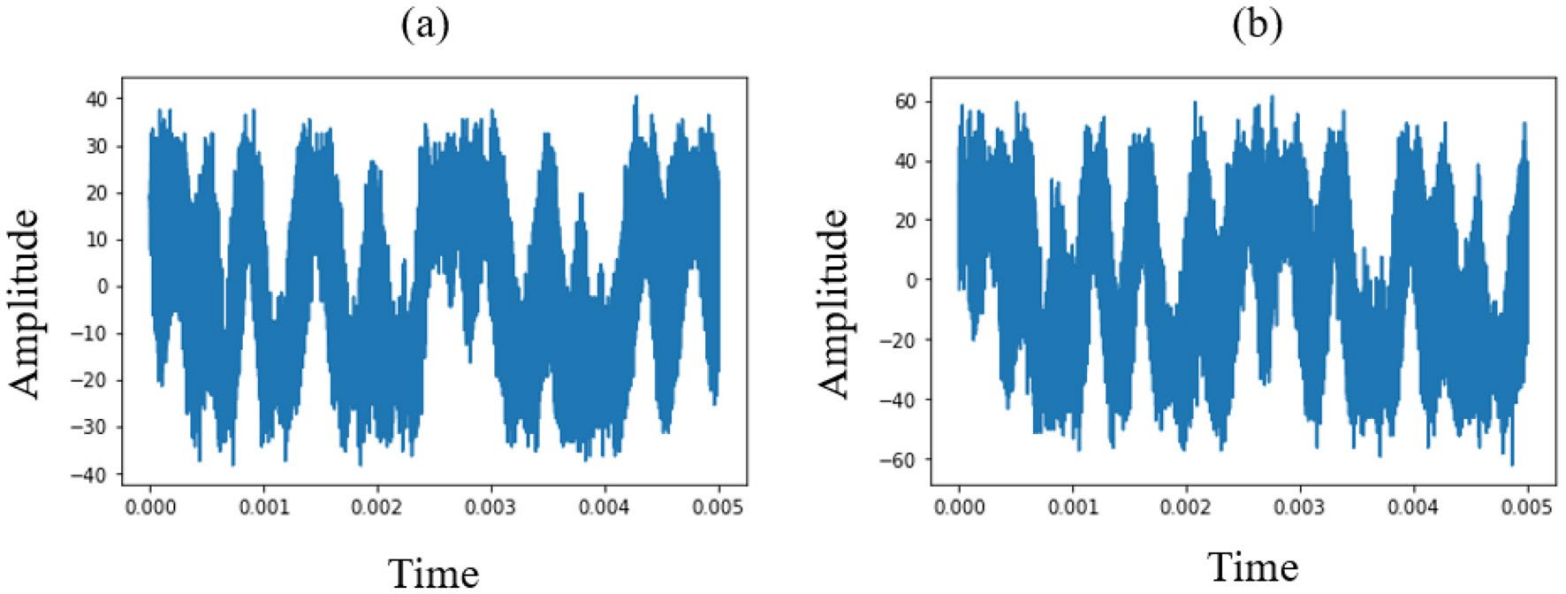
Time-domain envelopes of the WMT signal.

Figure 1 shows the time-domain envelops of the WMT signal. The CNR of both WMT signals is approximately 21 dB, but the amplitudes of both carrier signal and BGN differ. The amplitude of carrier signal (a) is − 90 dBm and (b) is − 86 dBm, and the amplitude of BGN (a) is − 111 dBm and (b) is − 107 dBm. Our proposed method estimates CNR by a single measurement and provides CNR values using these waveforms. As described earlier, both the WMT signal and BGN should be measured severally in these cases to calculate CNR. We investigated the acquiring features of time-domain waveforms using signal processing for machine learning.

We employed a low-cost software-defined radio (SDR) device as a receiver like a spectrum analyzer to provide an easier and low-cost method as much as possible. SDR is a radio communication system in which traditionally implemented components in hardware (e.g., mixers, filters, amplifiers, modulators/demodulators, and detectors) are instead implemented using software on a personal computer or an embedded system^13^. We estimated the CNR of WMT using time-domain waveform data measured by the SDR receiver and machine-learning techniques. We considered this approach as adequate for machine learning.

## Data collection and labeling

**Figure 2:**
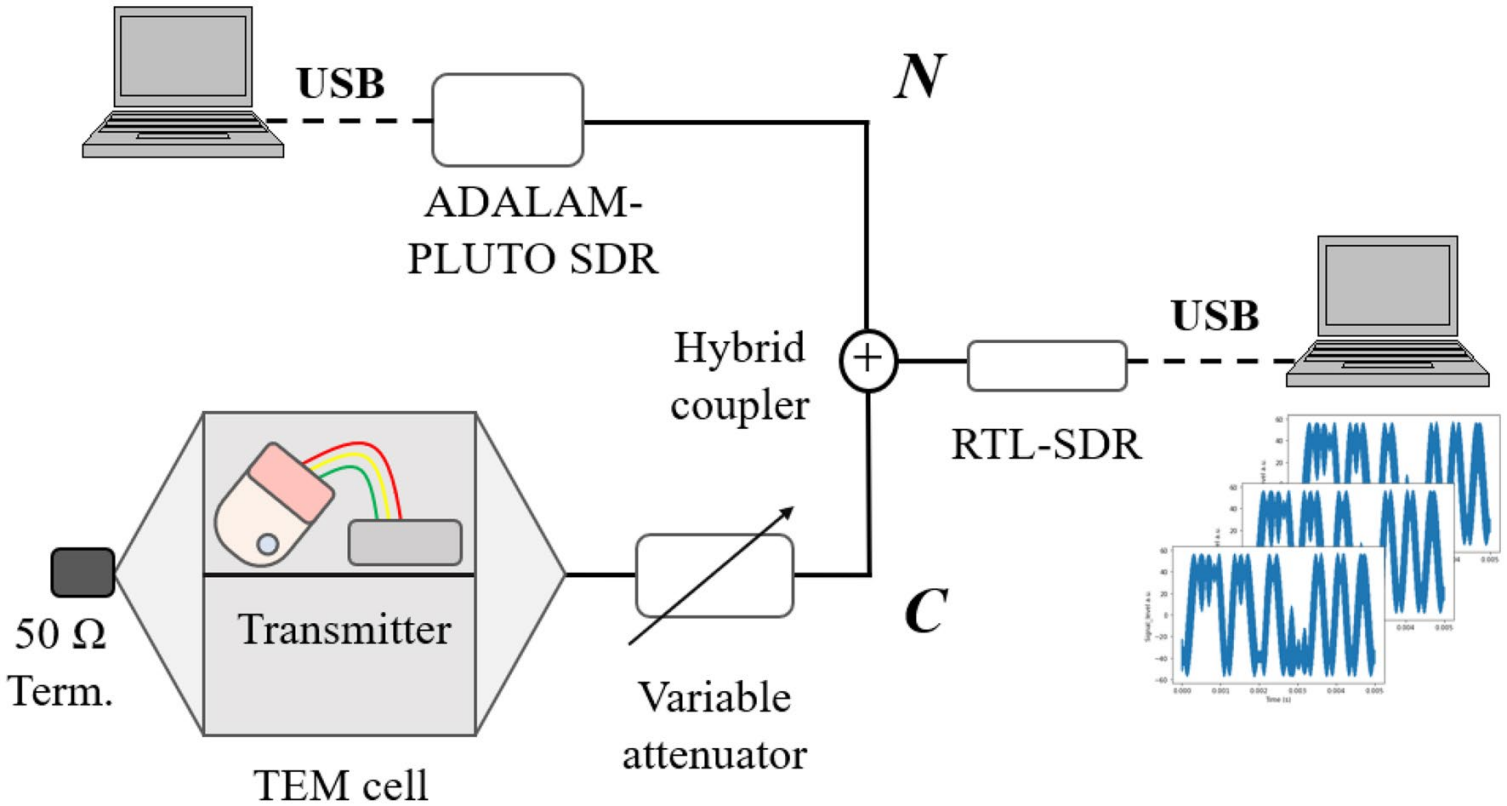
Measurement setup for WMT signals.

**Figure 3:**
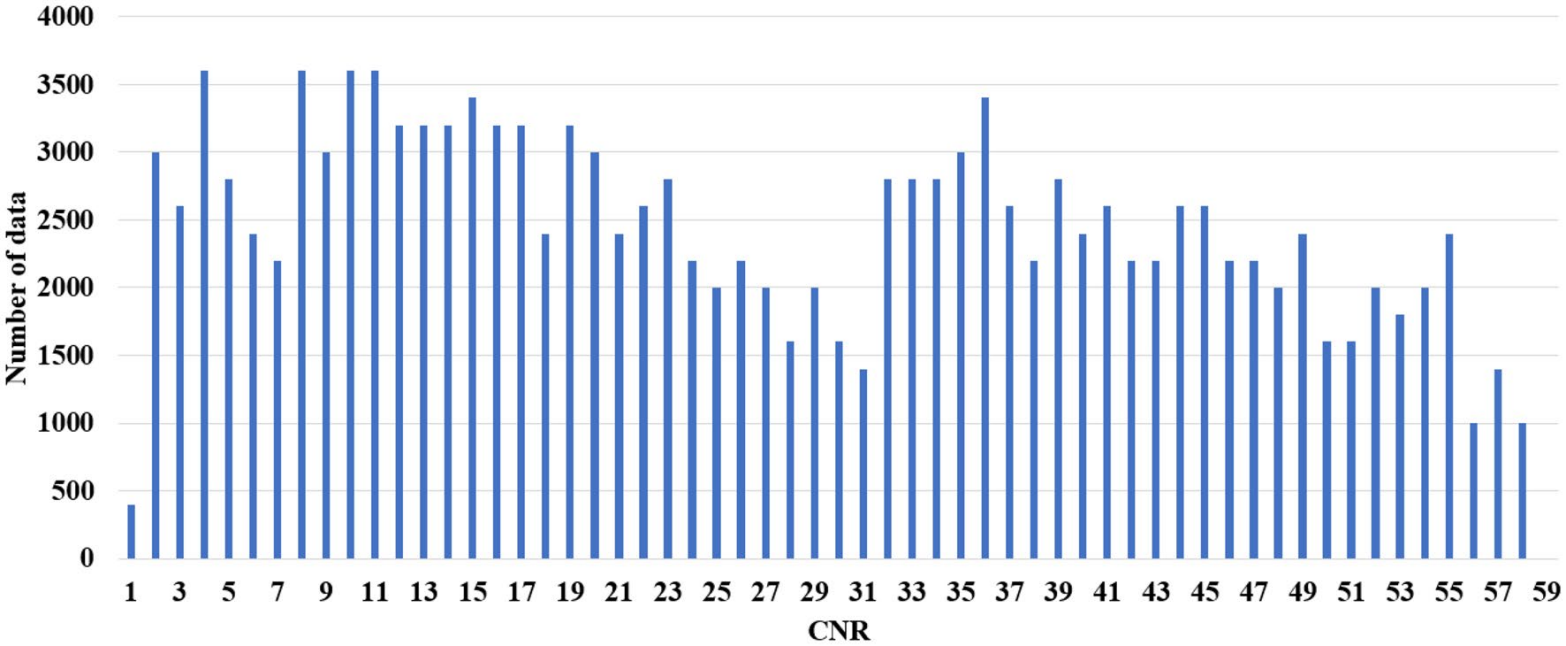
CNR distribution of the measured data.

We obtained the time-domain waveform of the WMT signal measured by a low-cost SDR receiver to structure datasets for machine-learning. We used a USB dongle-type SDR receiver (RTL2832U, RTL-SDR) with a resolution of 8-bit and a cost of approximately $30. This SDR was connected to a personal computer. Figure 2 shows the measurement setup. The WMT transmitter was placed in a transverse electromagnetic (TEM) cell to extract carrier signals only via the variable attenuator^14^. We used two types of transmitters made by different manufacturers: ZS-610P (Nihon-Kohden Co., Ltd.) and LX-8100 (Fukuda Denshi Co., Ltd.). The SDR transmitter (ADALM-Pluto, Analog Devices) connected with a personal computer for control was used as a signal generator which generated Gaussian noise in the 400 MHz band. We considered Gaussian noise to be BGN. According to a previous report, WMT employs a narrow band reception of 12.5 kHz; hence, most unwanted emissions generated from LED devices are band-limited to this bandwidth and are approximated as Gaussian noise^7^. Both the WMT signal and Gaussian noise were inputs to the SDR receiver through a hybrid coupler. The measurement was conducted at a measurement frequency of 429.325 MHz (using Japanese WMT channel number 3007^15^), the sample rate was 1 MHz, and the number of samples for one measurement was 2,000,000. Each measured data comprising 2,000,000 points was separated into 10,000 points. We obtained 708 conditions of data that were changed amplitudes of both WMT signal and Gaussian noise using a variable attenuator to replicate the various reception levels. Figure 3 shows the CNR distributions of the measured data. The CNR values ranged from 1 to 58 dB.

In this study, we used supervised learning, and labeled actual CNR values for each measured data. First, we measured the WMT signal and BGN power at each amplitude level using a real spectrum analyzer (RSA306B, Tektronix). The CNR was calculated from these WMT and BGN levels. We labeled a CNR of more than 30 dB or not to each data.

## Features, regressors, classifiers, and evaluation indexes for supervised machine-learning

**Figure 4:**
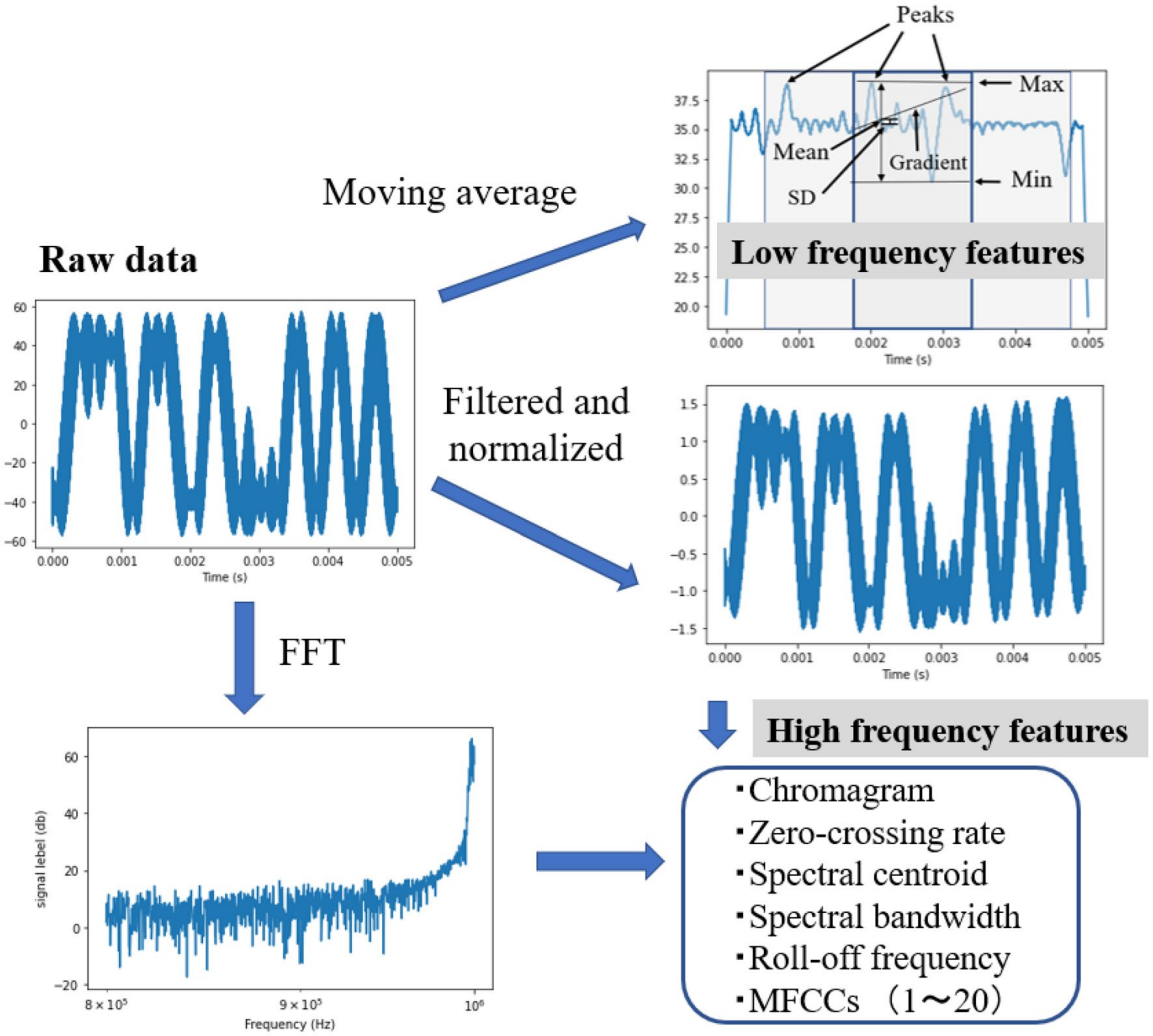
Schematics of signal processing for acquiring features.

In this study, we used 31-dimensional features calculated from the measured data by signal processing. Figure 4 shows the schematics of signal processing. First, we used simple moving averages with an interval of 300 points along the separated measured time-domain waveforms to detect envelope waves to obtain low-frequency features. We obtained the mean, standard deviation, minimum (Min), maximum (Max), and gradient calculated from envelope waves, which are between one-third and two-thirds of the total time. Moreover, the number of peaks of envelope waves between one-eighth and six-eighth of the time was calculated for each feature. In addition, we used the Butterworth filter, whose passband edge frequency was 10 Hz and stopband edge frequency was 40 Hz, to measure the time-domain waveforms for obtaining high-frequency features. Z-score normalization was used for this filtered data. Next, a fast Fourier transform was used to calculate the frequency spectra. We used librosa library, a Python module for audio and music processing, to obtain a chromagram, zero-crossing rate, spectral centroid, spectral bandwidth, roll-off frequency, and 1–20 coefficients of the Mel-frequency cepstral coefficients (MFCCs)^16,17^.

Candidates for the regressors and classifiers are k-nearest neighbor (KNN)^18^, logistic regression (LR, classifier only)^19^, decision tree (DT, classifier only)^20^, random forest (RF)^21^, support vector machine (SVM)^22^, gradient boosting regression tree (GBRT) and decision tree (GBDT)^23^, ensemble algorithm (Adaboost)^24^, and two-layer neural network (NN)^25^. We tuned hyper parameters in these regressors and classifiers; number of neighbors in KNN, maximum depth, maximum leaf nodes and minimum sample leaf in DT, number of trees and maximum depth in RF and GBRT, the radius basis function kernel (gamma) and regularization parameter (*C*) in SVM, learning rate and number of boost round in GBDT, number of trees, maximum depth and learning rate in AdaBoost and epoch size, batch size and learning rate in NN. After which we selected the best parameters.

To avoid overtraining, we employed a 5-segment cross-validation for regression analysis and Stratified Group K-Fold (K = 5) for classification. In regression analysis, ${R}^{2}$ score, which is the coefficient of determination and mean absolute error (MAE), was used as a criterion for evaluating the performance of supervised learning. In contrast, accuracy and recall were used in the classification instead. Generally, accuracy, true positive rate (TPR), true negative rate (TNR), false-negative rate (FNR), and false-positive rate (FPR) are used as criteria for evaluating the performance of supervised machine-learning algorithms, as defined by the following formulas:

1 $$\mathrm{Accuracy }= (\mathrm{TP }+\mathrm{ TN}) / (\mathrm{TP }+\mathrm{ FP }+\mathrm{ TN }+\mathrm{ FN})$$

2 $$\mathrm{TPR }=\mathrm{ Recall }=\mathrm{ TP }/ (\mathrm{TP }+\mathrm{ FN})$$

3 $$\mathrm{TNR }=\mathrm{ Precision }=\mathrm{ TN }/ (\mathrm{FP }+\mathrm{ TN})$$

4 $$\mathrm{FNR }=\mathrm{ FN }/ (\mathrm{TP }+\mathrm{ FN})$$

5 $$\mathrm{FPR }=\mathrm{ FP }/ (\mathrm{FP }+\mathrm{ TN})$$

where TP, TN, FP, and FN are the number of correct predictions for positive and negative subjects, and the number of incorrect predictions for positive and negative subjects, respectively. To evaluate these evaluation indices, we divided the measured data of each subject into training data (90%) for supervised learning and test data (10%) for evaluation.

The training and validations were executed using Windows 10 Pro personal computer (CPU: Intel Core i9, Memory: 192 GB, SSD: 1 TB and GPU: NVIDA GeForce RTX 2080Ti). Following Python libraries were used to construct machine-learning models: scikit-learn for KNN, LR, DT, RF, SVM and GBRT/DT, Keras and Tensorflow for NN, and XGBoost for Adaboost.

## Experimental results

**Figure 5:**
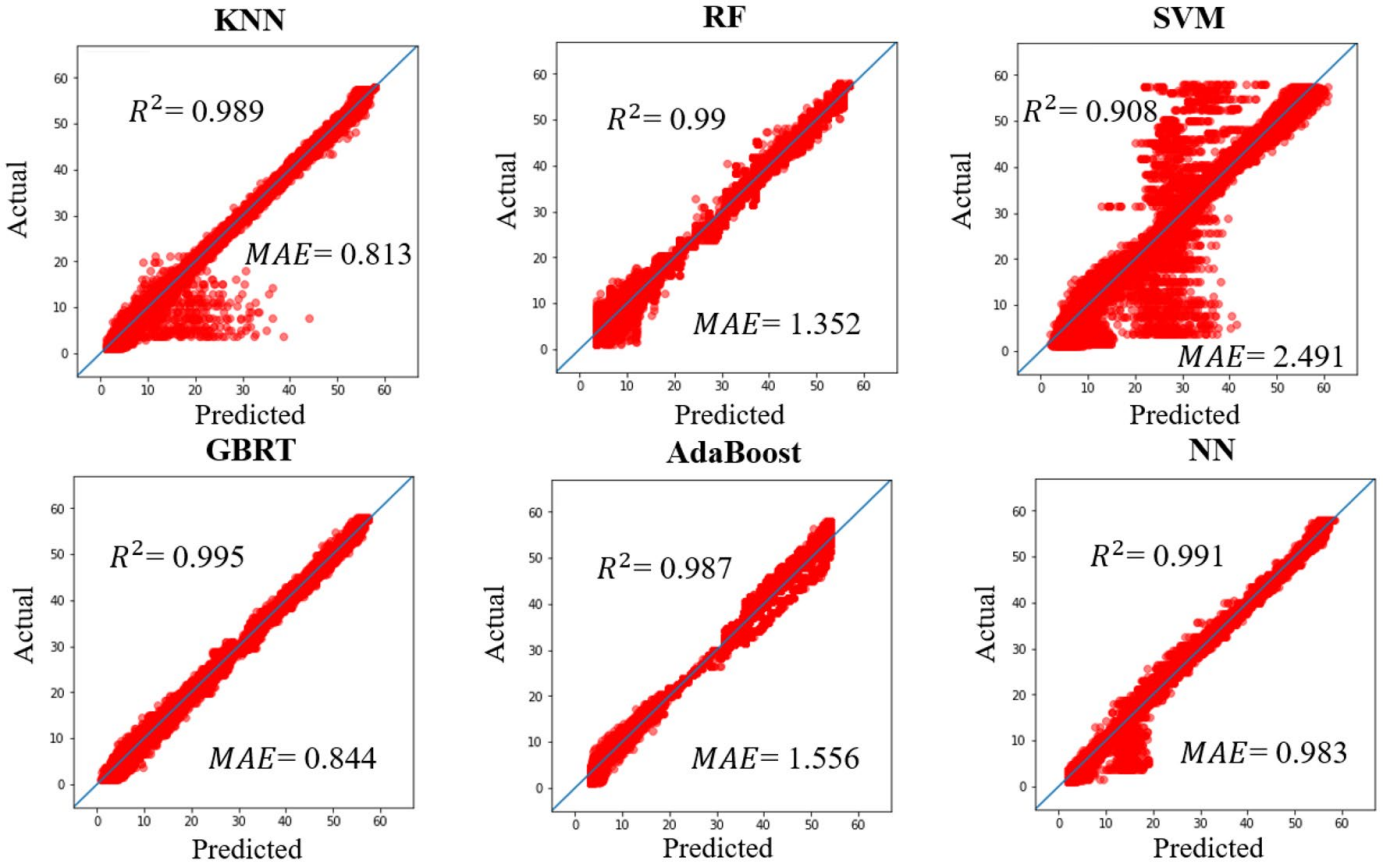
Results of the regression analysis.

Figure 5 shows the ${R}^{2}$ score and MAE for each regressor. All regressors achieved a ${R}^{2}$ score exceeding 0.9, but the MAE ranged from 0.844 to 2.491. The SVM regressor had large variations of low to high level of CNR. The KNN regressor achieved high ${R}^{2}$ score and MAE was the best in all regressors, but the estimation errors occurred on low level CNR. The RF, GBRT, Adaboost and NN regressors had good linearity, of which the GBRT regressor was the highest with ${R}^{2}$ score of 0.995 and MAE of 0.844.

**Figure 6:**
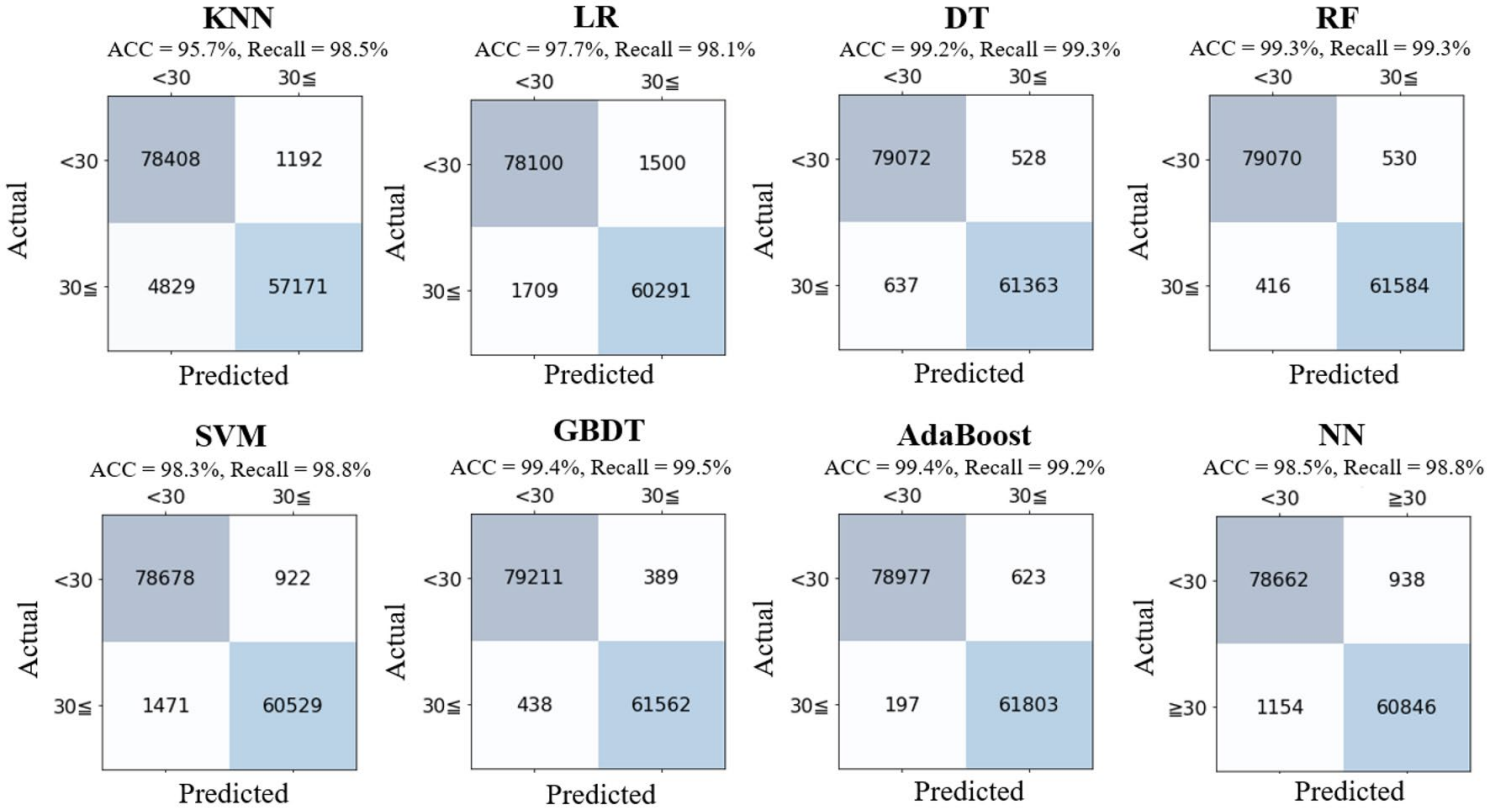
Results of the classification analysis.

Figure 6 shows the confusion matrix for each classifier and depicts accuracy (ACC) and recall. All classifiers have similar results and achieved a higher accuracy exceeding 95% and a high recall exceeding 98%, of which the GBDT classifier was the best (ACC = 99.4%, recall = 99.5%). From the point of view of the risk management, overlooking of low level CNR may invoke poor reception of WMT. It is desirable to have a lower value of FN, hence, making GBDT the best classifier.

## Adequacy and limitations

The CNR of WMT (3–50 dB) was reported at the Japanese University Hospital ward^28^ and 6–61 dB at the simulated ward of the university department^29^. Our training dataset approximately covers these CNR values. However, we reproduced various conditions of CNR obtained by varying the amplitude of both the WMT signal and BGN, indicating that our dataset covers cases of good reception with no EMI factor and the worst conditions of BGN increase.

In this study, we used a low-performance SDR device as a receiver to measure WMT signals. However, this SDR can capture a low amplitude signal of approximately − 120 dBm; hence, making it sufficient to measure WMT signals at actual hospitals.

In our learning model, the GBRT regressor was the best and could estimate CNR within the error range of 1%. This high-precision estimator can provide a detailed survey of the electromagnetic environment in hospitals. For example, it could be useful for maintenance before or after replacing radio-receiving equipment, such as antennas and amplifiers. Furthermore, the GBDT classifier, which was our model’s best accuracy, was sufficient in determining whether the CNR exceeded 30 dB. This model is promising for screening poor reception of WMT for periodic investigation in hospitals. Notably, managing the electromagnetic environment is important for the safe operation of medical devices, including WMT^30^. However, most hospitals have generally been short staffed in the management of EMC. Our learning models are efficient in complementing the lack of skills, manpower, and specific instruments in clinical settings.

In this study, we targeted only the Japanese WMT, which uses the frequency of 400 MHz band. However, every country or region has different allocated frequency bands for WMT. For instance, Japan uses 400 MHz bands, while the US and EU use 600 MHz and 1400 MHz bands respectively^15,31^. However, the specifications of both Japanese and US or EU WMT systems are almost the same: the transmitting power (1 mW), modulating scheme (frequency shift keying), and channel bandwidth (12.5 kHz). As the carrier frequency bands of the US and EU WMTs differ from that of Japan, but with the communication system being the same, we can acquire time-domain waveforms using the same measurement and can estimate CNR using our learning model.

Moreover, our datasets were collected from measurements by wired setting, but WMT signals are propagated in the air in actual settings. However, we cannot change the setting of the output power of the WMT transmitter in the body. But to reproduce and capture the various conditions of signal levels, we selected a wired setting that uses a TEM cell and variable attenuator. In clinical settings, WMT signals may vary due to the following reasons: reflection and diffraction on the wall, celling and flooring and the patient’s body movement. However, as described earlier, our dataset covers low to high level CNR. If the WMT signal was varied broadly due to these effects, we can use the worst value as CNR.

We used Gaussian noise to increase BGN. In our previous study, we approximated most LED noise as Gaussian owing to band-limitation effect of the WMT receiver^7^. We intended to increase BGN as the effect of LED noise on WMT. However, some LED noise has sequential or impulsive characteristics, with a cycle of 50 Hz derived from the Japanese mains power supply, even in the band-limited reception^32^. This sequential noise causes approximately 3 dB worth of receiver sensitivity compared to LED noise, which can be approximated as Gaussian noise. On the other hand, by focusing on the highest position of the noise amplitude and broadening it, we can approximate this position as Gaussian noise even if the measured waveform has a sequential or impulsive nature. Therefore, our measured data also covers the increase in BGN caused by sequential and impulsive characteristic noise.

## Discussion

In this study, we presented novel estimation technique for the CNR of WMT using machine-learning. Similar research in the estimating of signal to noise ratio (SNR) of wireless signals has been reported. Kojima reported that SNR was estimated by artificial NN, but this estimation was targeted to broad band communication such as wireless local area network of OFDM signal with a bandwidth of 20 MHz^26^. On the other hand, we targeted narrow band communication of WMT with a bandwidth of 12.5 kHz. Xie reported other approach of SNR estimation that uses constellation diagrams of wireless signals and convolutional NN^27^. However, we used time domain waveform of WMT signals instead of constellation diagrams for estimating CNR. Therefore, our approach is different from these past studies.

The major features of our method are the ease of estimation and the low cost in estimating CNR of WMT. We simultaneously measured and automatically estimated CNR at high accuracy. No prior skills or knowledge on electromagnetic waves and wireless communications are required. Moreover, we used a less expensive SDR dongle that costs approximately $30 in substitution for a spectrum analyzer to measure the electromagnetic environment. The cost of a stand-alone spectrum analyzer usually exceeds $1000; that of the USB-connected type exceeds $400, but it is necessary to personal computers with a certain level of specification. The SDR dongle used in this study did not require a high-performance computer. Using a single-board computer such as Raspberry Pi as a control device, the SDR dongle was satisfactory for the measurement, and the total cost of our system was estimated as $100–120. A single-board computer with a mounted graphic processing unit, such as NVIDIA Jetson Nano, is suitable for operating our learning model. As an alternative approach, our machine-learning models can be installed in the WMT receiver. In this case, no other computer is needed for connecting SDR or spectrum analyzers. However, existing radio-receiving equipment was used for the measurement. For future work, we propose installing our learning models on both single-board computers and WMT receivers.

## Conclusion

We investigated a machine-learning model for estimating the CNR of WMT and found that GBRT was the best regressor and could estimate CNR within the error range of 1%. Also, GBDT was the best to classify whether the CNR exceeded 30 dB. Our proposed method evaluates the electromagnetic environment using a simplified, automated, and low-cost approach.

## Data Availability

The datasets used and/or analyzed during the current study available from the corresponding author on reasonable request.
